# The analytical potential of the dry blood spot technique for pesticide biomonitoring — a review

**DOI:** 10.1007/s10661-025-14930-6

**Published:** 2025-12-23

**Authors:** Kamila Komajda, Grzegorz Teresiński

**Affiliations:** Chair and Department of Forensic Medicine, Medical University of Lublin, Lublin, Poland

**Keywords:** Microsampling, DBS, Biocides, Agricultural chemicals, Exposure

## Abstract

Microsampling is a technique involving the collection of a small amount of biological material in a volume of several to dozens of microliters. The most common material collected is blood from a fingertip. Following a finger prick, dry blood spots are formed after being transferred to a carrier. The method is microinvasive and causes little discomfort during the collection. The preservation of blood in the form of dry spots seems to be an ideal tool for pesticide exposure assessment in the general population and occupationally exposed groups. Populations in agricultural areas are constantly exposed to environmental xenobiotics, including a wide range of pesticides and other pollutants. This technique can be a practical way to assess pesticide exposure, particularly in rural areas or places where whole blood collection is difficult. The issue has generated considerable scientific debate, primarily due to challenges associated with the analysis of small blood spots of unknown volume and the influence of the haematocrit (HCT) effect. The use of dry blood spots can significantly contribute to the expansion of the scale of pesticide biomonitoring, which will allow for the quick and effective identification of individuals exposed to their effects. The technique of microsampling and analysis of DBS indicate great potential in the assessment of pesticide exposure due to its simplicity of specimen collection, minimal invasiveness, and the possibility of storing and transporting the material without the use of specialized conditions. Clinical trial number: not applicable.

## Introduction

Microsampling is a technique involving the collection of a small amount of biological material, most frequently blood or urine, in a volume ranging from a few to several dozen microlitres (Thangavelu et al., 2023). The first reports of successful use of microsampling for analytical purposes date back to 1913 when Ivar Christian Bang utilized cellulose cards as a carrier for the quantitative determination of glucose from the venous blood of farm rabbits (Malsagova et al., 2020). Since 1924, modifications to cellulose paper have allowed the application of the dried blood spot (DBS) technique in serological tests, including the diagnosis of syphilis in adults and children, smallpox, measles as well as polio, parainfluenza, and respiratory syncytial virus (RSV) (Grüner et al., 2015). In 1963, the application of DBS for screening was popularized due to Guthrie and Susi (1963), who utilized it to screen newborns with phenylketonuria. Analysis of xenobiotics secured in the form of DBS began to gain momentum in the 1990s. Studies have shown that microsampling can be used to determine a wide range of analytes, including hormones, drugs, and their metabolites; thus, it constitutes a valuable tool in both clinical and forensic toxicology studies (Lehmann et al., 2013; Malsagova et al., 2020; Stove et al., 2012). The use of DBS is currently an interesting alternative to whole blood analysis.

## Use of DBS in clinical toxicology

DBS in clinical toxicology is mainly used during therapeutic drug monitoring (TDM) (Cafaro et al., 2023; Spooner et al., 2009; Wilhelm et al., 2014). The growing interest in DBS is primarily associated with the benefits provided by the microsampling technique. Having read the instructions, patients can collect blood themselves. Moreover, the volume of blood collected is small. A review of the scientific literature confirmed the use of DBS during therapy for monitoring treatment for various classes of drugs, including anticonvulsants, antiretrovirals, immunosuppressants, antimalarials, antiepileptics, cardiology, antibiotics, analgesics, and antidepressants (Antunes et al., 2016; Edelbroek et al., 2009; Lawson et al., 2013; Shah et al., 2013; Vnučec Popov et al., 2013). A study by Orleni et al. (2025) concluded that DBS specimens are a reliable alternative to plasma samples for TDM of imatinib and its metabolite, norimatinib. The high–performance liquid chromatography coupled to a spectrophotometer method (HPLC–UV) developed by Chaudhari et al. (2023) confirmed that DBS can be successfully used to monitor ceftriaxone concentrations in the neonatal population.

## Application of DBS in forensic toxicology — intravital and post–mortem analyses

Currently, DBS have been used for qualitative and quantitative analysis of many narcotic, psychotropic, and psychoactive substances. A cohort study by Guo et al. (2023) demonstrated the usefulness of the microsampling technique in detecting 425 narcotic and psychotropic substances. The substances, with individual exceptions, showed stability from 3 to 5 years at room temperature. The developed procedure was used to analyse 105 real samples secured from individuals suspected of drug poisoning (C. Guo et al., 2023). The conducted studies confirmed the usefulness of DBS for the identification of cocaine and its metabolites (Ambach & Stove, 2019; de Lima Feltraco Lizot et al., 2019). Furthermore, DBS samples secured from clothing were successfully used for the toxicological analysis of synthetic cathinones and their metabolites (Wang et al., 2021). In the study conducted by Komajda and Przygodzka (), a method for isolating xenobiotics used in drug-facilitated sexual assault (DFSA) from dry blood spots set on clothing material was developed. Analysis of DBS taken from athletes before or after competition can provide information on the temporal presence or absence of prohibited substances during sports competitions (Garzinsky et al., 2023; Tretzel et al., 2015). Currently, the method has been developed for the isolation and qualitative and quantitative determination of 26 substances representing eight different classes of performance–enhancing drugs, i.e. hormonal and metabolic modulators, anabolic agents, stimulants, beta–blockers, diuretics, β2–agonists, glucocorticosteroids, and cannabinoids (Thevis et al., 2023). The method developed by Tegner et al. (2022) was successfully applied to the analysis of capillary and venous blood collected from 20 volunteers who consumed ethanol. A correlation between the results for DBS from capillary blood and venous blood was demonstrated. This study confirmed the usefulness of the microsampling technique for determining markers of acute alcohol consumption, i.e. ethyl glucuronide (EtG) and ethyl sulfate (EtS).

The DBS technique has been used to determine, among others, psychotropic drugs from post–mortem blood (Nishio et al., 2023) such as citalopram, duloxetine, mirtazapine, olanzapine, paroxetine, quetiapine, sertraline, and Z-drugs. The method developed by Dubois-Chabert et al. (2025) was used to analyse 102 post–mortem cases, demonstrating its effectiveness in detecting various compounds with high sensitivity. The results of a study conducted in 2019 indicated a good correlation between DBS samples and post–mortem whole blood samples for benzodiazepines (Moretti et al., 2019). In addition, most compounds remained stable for three months at room temperature. The protocol developed by Hakim et al. (2025) emphasized that DBS can be an alternative to post–mortem toxicological analysis, particularly in situations where conventional blood sampling is difficult or impossible. The method developed and validated by Sadler Simões et al. (2018) enabled the identification of eleven illegal substances from DBS secured from living and deceased persons. During DBS analysis, amphetamine and methamphetamine, cocaine and its metabolites, opioids as well as ketamine, mescaline, and tramadol were also isolated from post–mortem material (Meikopoulos et al., 2024).

## Materials and methods

The search criteria were limited to journal articles, books, and scientific reports published in English. The scientific literature was searched in databases such as “PubMed”, “Web of Science”, “Scopus”, and “Google Scholar”. The Boolean operators “AND”, “OR”, and “AND/OR” were used. The phrases introduced were as follows: (“dry”, AND “spots”, AND “blood”), (“dry”, AND “spots”, AND “blood”, AND “pesticides”), (“DBS”, AND “pesticides”), (“Whatman 903” AND “pesticides” AND “HPLC**–**MS/MS”), (“blood”, OR “urine” AND “pesticides”, AND “HPLC”), (“toxicology” AND/OR “clinical” AND/OR “forensic” AND “DBS”), (“DMT” AND “DBS”), (“DBS” AND “HPLC**–**MS/MS” OR “GC**–**MS” AND “pesticides”), (“DBS” AND “matrix effect” AND/OR “chromatographic effect”), (“DBS” AND “post mortem” AND “haematocrit effect”), (“DBS” AND “hemolysis”), (“microsampling” AND “technique”).

Eligibility criteria included studies that focused on the use of dried blood spots in pesticide analysis, toxicological applications, and related microsampling techniques. Publications not available in English, abstracts without full text, conference posters, and studies unrelated to dry blood spots or pesticide exposure were excluded.

## Results

### DBS collection, transport, and storage for toxicological analyses

**Fig. 1:**
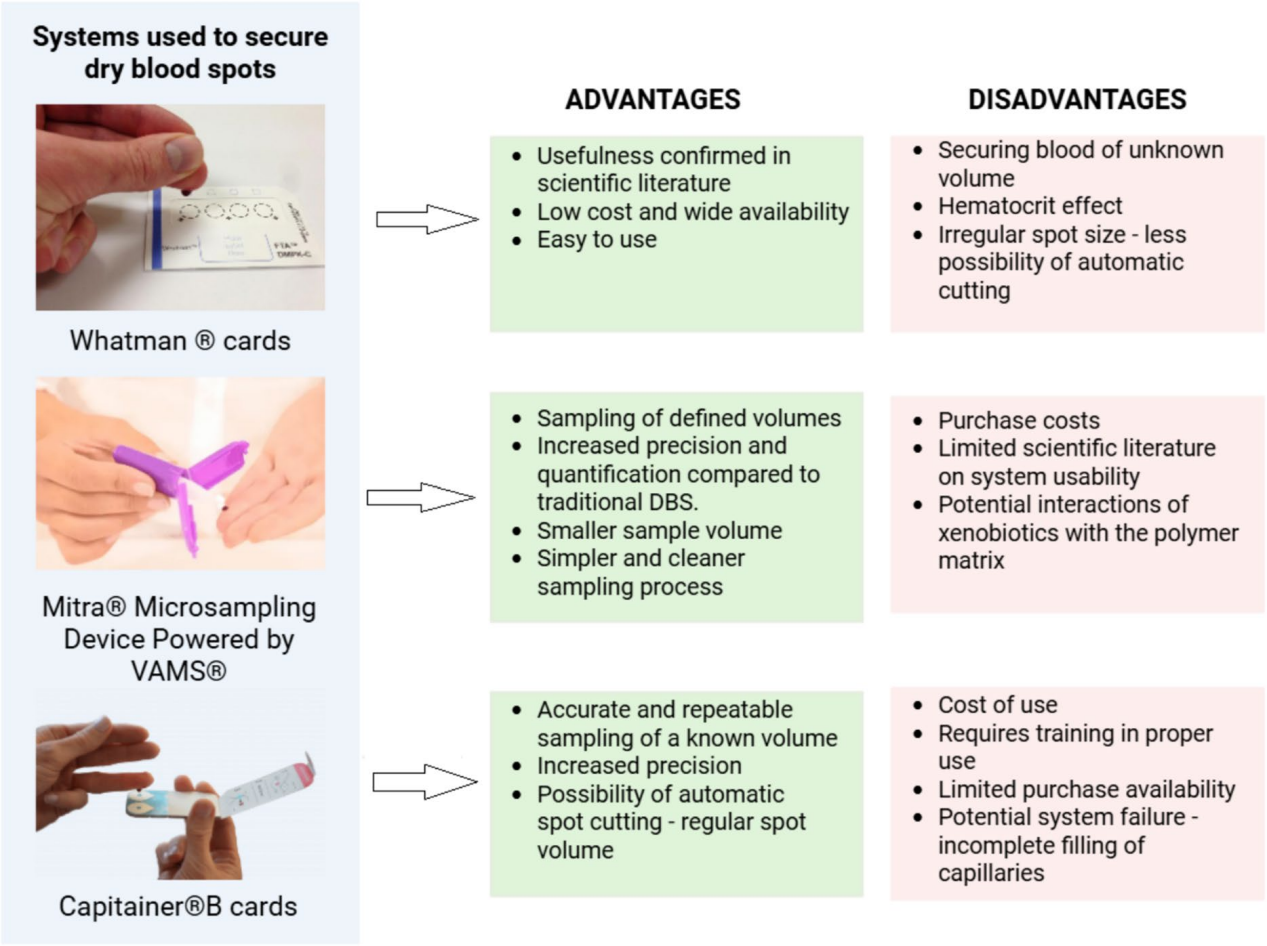
Comparison of advantages and disadvantages of systems used to protect DBS

DBS collection is a relatively simple and minimally invasive procedure that does not require the presence of trained medical personnel. Currently, there are several types of media available on the market for securing DBS. First of all, Whatman 903® are classic cards used for newborn screening and biomarker analyses. However, media with an absorption tip are increasingly appearing on the market, allowing for the collection of a controlled volume of blood, e.g. Neoteryx Mitra®. Using volumetric absorption microsampling (VAMS), it is possible to collect an exact volume of blood thanks to a special polymer tip applied to the puncture site (De Kesel et al., 2015; de Sá e Silva et al., 2023). An interesting alternative is the application of modified cards with external dispensers or capillary applicators, e.g. Hemaxis DB10 (Capitainer®) (Orleni et al., 2025). Figure 1 shows a comparison of systems used in the microsampling technique. Prior to the collection, the fingertip should be disinfected and cleaned to be free from any remaining dust or dirt. The first drop formed should be wiped off with sterile gauze. Each subsequent drop should fall onto the carrier spontaneously without the direct contact of the finger with the paper or capillary forces pulled by the polymer tip. Blood specimens can also be applied to the filter paper using standard capillaries of known volume used for gasometric testing. This use will minimize the error resulting from the variable volume of formed blood drops (Barr et al., 2021). In the case of securing blood from a deceased person, the desired volume of blood should be transferred to the biological material carrier using a pipette. The recommended drying time of blood spots for toxicological analyses remains a controversial issue. It seems optimal to dry samples for 2 to 4 h or leave them overnight at room temperature (Basavaraju & Pitman, 2014; Malsagova et al., 2020). Samples stored in a high humidity environment may require a longer drying time compared to samples stored in a sunny environment. After drying, it is recommended to store samples at 2 to 4 °C or freeze them to minimize the risk of xenobiotic degradation (Barr et al., 2021; Batterman & Chernyak, 2014). Cards or other media should be stored in tightly closed ziplock bags with moisture–absorbing sachets (Crimmins et al., 2020; Lim, 2018; Niemiec, 2021). As with any biological sample, repeated freeze–thaw cycles should be avoided if samples are stored in a freezer. Stained media should be separated by a clean sheet of paper or stored in separate ziplock bags to avoid cross–contamination during transport or storage (Barr et al., 2021).

## Advantages and disadvantages of the microsampling technique

Venous blood collection is relatively invasive and requires the presence of authorized medical personnel (Jacobson et al., 2023). The main advantages of the microsampling technique include relatively simple collection of material, its shipment, and storage. The method is minimally invasive and associated with minimal discomfort during sample collection. Storing xenobiotics protected in the form of DBS slows down their degradation, thanks to which these samples can be analysed with high accuracy after a significant lapse of time (Cui et al., 2024; Majda et al., 2019; Prentice et al., 2013). The DBS technique does not require specialized procedures for sample preservation and enables self-collection, which constitutes a significant advantage in the context of TDM or surveillance of individuals with substance use disorders (Jacobson et al., 2023). The risk of infection with pathogens during DBS processing is negligible — viruses are inactivated during the spots drying (Niemiec, 2021). Storing samples secured in the form of dry spots is much simpler than storing whole blood and also generates lower costs. The material in the form of DBS is suitable for biobanking and retrospective analyses (Lim, 2018). Blood preservation in the form of DBS seems to be an ideal solution for measuring the level of chemical substances or their metabolites in biological samples, due to the collection simplicity and possibility of archiving. In order to use the microsampling technique for the analysis of trace substances, it is necessary, first of all, to standardize the procedures for collecting and storing samples. Due to the variety of xenobiotics analysed, these procedures may differ.

The main analytical challenge in blood spot analysis is the small and variable volume of the analysed spot (Komajda & Przygodzka, 2025). DBS analysis requires a preliminary sample purification procedure, e.g. by liquid–liquid extraction or solid–phase extraction to reduce the matrix effect and obtain the desired detection limit for the analytes. Extraction of xenobiotics from DBS can be a time–consuming process; the selection of the extraction solvent largely depends on the chemical structure of the analysed group of compounds. The preferred methods used for the determination of xenobiotics in the form of DBS are high–performance liquid chromatography coupled with mass spectrometry (Chen et al., 2024; Luginbühl & Gaugler, 2020; Rao, 2014; Roberts et al., 2025; Wagner et al., 2016; Zakaria et al., 2016).

### Estimation of blood spot volume

One of the major problems associated with the use of DBS for trace analysis is the lack of standardized procedures for accurate measurement of the preserved blood spot volume after finger prick. Incorrect estimation of spot size can affect the accuracy of the analysis of xenobiotics present in DBS. This problem is associated with card–based media, e.g. the standard Whatman 903® cards. Several strategies have been proposed to minimize the problems associated with the determination of the spot volume. The recommended technique is to apply whole blood to filter paper by means of capillaries delivering a fixed volume of blood. Facilitation of the collection of a specific volume is based primarily on modifications to the cards or blood collection devices (Baillargeon et al., 2022). DBS collection systems, i.e. Mitra® (Wikström et al., 2023) and Capitainer® (Velghe & Stove, 2018), enable easy collection of DBS samples with precise volume. The challenge is presented by both new and archival spots of unknown volume, secured on standard Whatman 903® cards (Majda et al., 2019). Endogenous substances and their metabolites can be used to estimate blood spot volume. A study by Lee et al. (2023) showed that endogenous amino acids, i.e. L–isoleucine and L–phenylalanine, can be used as internal standards to estimate the volume of DBS samples. The analysis showed that the concentration of these amino acids increased as the volume of spotted blood increased. The Postcolumn Infused–Internal Standard strategy (PCI–IS) proposed by Liao et al. (2016) was based on the DBS volume estimation by total salt content (mainly sodium and its counteranions) in the blood and the ion suppression effect. By constructing a calibration curve using reference blood samples with known volumes, the method allowed for accurate estimation and correction of blood volume changes. Blood volume can also be estimated based on the DBS diameter, blood specific gravity, and the ratio of fresh blood to dehydrated blood volume (Seo & Batterman, 2024b). Currently, there is no reference method for accurate determination of the volume of blood preserved as a dry spot. This is the main challenge encountered during the analysis of archival blood spots (Majda et al., 2019).

### The HCT effect, haemolysis, and chromatographic effect

The HCT effect, along with the estimation of the volume of blood applied to the card, constitutes a major obstacle to the routine use of the DBS technique in toxicological analyses. HCT represents the proportion of erythrocyte volume relative to the total volume of whole blood, typically expressed as a percentage. The HCT value directly reflects the viscosity of blood that affects the diffusion of blood spotted onto the card carrier. During the interaction of blood with chromatographic paper, a slow spreading of the spot in a circle can be observed, described as the chromatographic effect in the literature (Barr et al., 2021). The chromatographic effect modifies the way the analyte is distributed on the card. Depending on the structure of the analyte, the chromatographic effect can either bring the analyte to the edge of the spot or concentrate it in the centre. Elevated HCT levels increase blood viscosity, which in turn adversely affects the distribution of the blood spot on the collection card, leading to a reduced spot size. The spot obtained from a blood specimen with a lower HCT and lower blood viscosity is usually larger (Ostler et al., 2014). DBS samples obtained from blood with a low HCT form large spots in which the analyte can be distributed evenly over the entire spot field. In the case of a small spot diameter, the analysed xenobiotics can be concentrated in the centre. These differences are, therefore, an important source of measurement error. However, the study by Holub et al. (2006) showed that analyte concentrations in DBS are higher for samples taken from individuals with higher HCT and correspondingly lower for low HCT, regardless of the site of excision of the DBS fragment.

In the case of post–mortem blood samples, they may show different HCT levels due to physiological changes occurring after death, e.g. haemolysis. Haemolysis leads to a reduction in HCT levels as a result of the lysis and consequent decrease in the number of erythrocytes. These changes may potentially affect the diffusion of the analyte in the card and the quantitative analysis of the xenobiotic. In the research by Bossi et al. (2025) into venous blood collected from living volunteers, extraction protocols for five extractants were developed and optimized by means of three commercially available microsampling devices — Whatman 903® Protein Saver Cards, Capitainer® B, and Telimmune DUO Plasma Separation Cards. The authors of the study suggested that the phenomenon of material haemolysis that may result from direct DBS collection and spot extraction may interfere with metabolomic and lipidomic analysis by suppression or amplification of the signal for the analysed ions. Although this study does not directly investigate post–mortem blood, it provides valuable suggestions regarding the effect of haemolysis on ion identification. In the analysis of DBS collected post–mortem, the potential effect of haemolysis on HCT levels should be taken into consideration.

Mathematical correction methods can be used to eliminate the HCT effect. These corrections are intended to estimate the analyte concentration in DBS samples based on the subject’s HCT level (Cheng et al., 2023). HCT correction methods are mainly based on mathematical formulae and calculated correction factors (Daousani et al., 2019). Correction factors for specific analytes can be determined from the simple regression equation for a series of calibration samples with different HCT levels. To apply the correction, a reference HCT value must be determined at which it is assumed that the analyte concentrations are consistent across all samples (Cheng et al., 2023). For the determined value, it can be assumed that the analytes are distributed evenly in the dry spot regardless of the location of the smaller fragment (punch). This allows for comparison of results between samples that differ in HCT. Although the authors of the study by Cheng et al. (2023) do not indicate a single reference HCT value in their publication, their approach allows for the assessment of the effect of different HCT levels and the correction of the analysis results depending on the actual HCT samples. Another strategy for the elimination of the HCT effect and uneven distribution of analytes in blood on the card involves the application of the entire blood spot instead of individual punches punched or cut out of the card (Malsagova et al., 2020; McClendon-Weary et al., 2020). In the study by Richardson et al. (2018), a method of HCT estimation was used based on haemoglobin measurement by means of sodium lauryl sulfate. The method showed good correlation with traditional measurement of HCT samples allowing simple and rapid estimation of predicted HCT in DBS.

The analysis of single spots can result in false quantifications due to non–uniform distribution of the analyte in the spot (Majda et al., 2019). In the case of only a fragment of the spot being available, the aforementioned correction for the HCT effect should be performed. The correct determination of the analysed samples is subject to the HCT effect, and its elimination is a critical initial step in the development of routine methods for DBS analysis (Alsous et al., 2020; Cheng et al., 2023).

## The use of DBS for pesticide determination

**Fig. 2:**
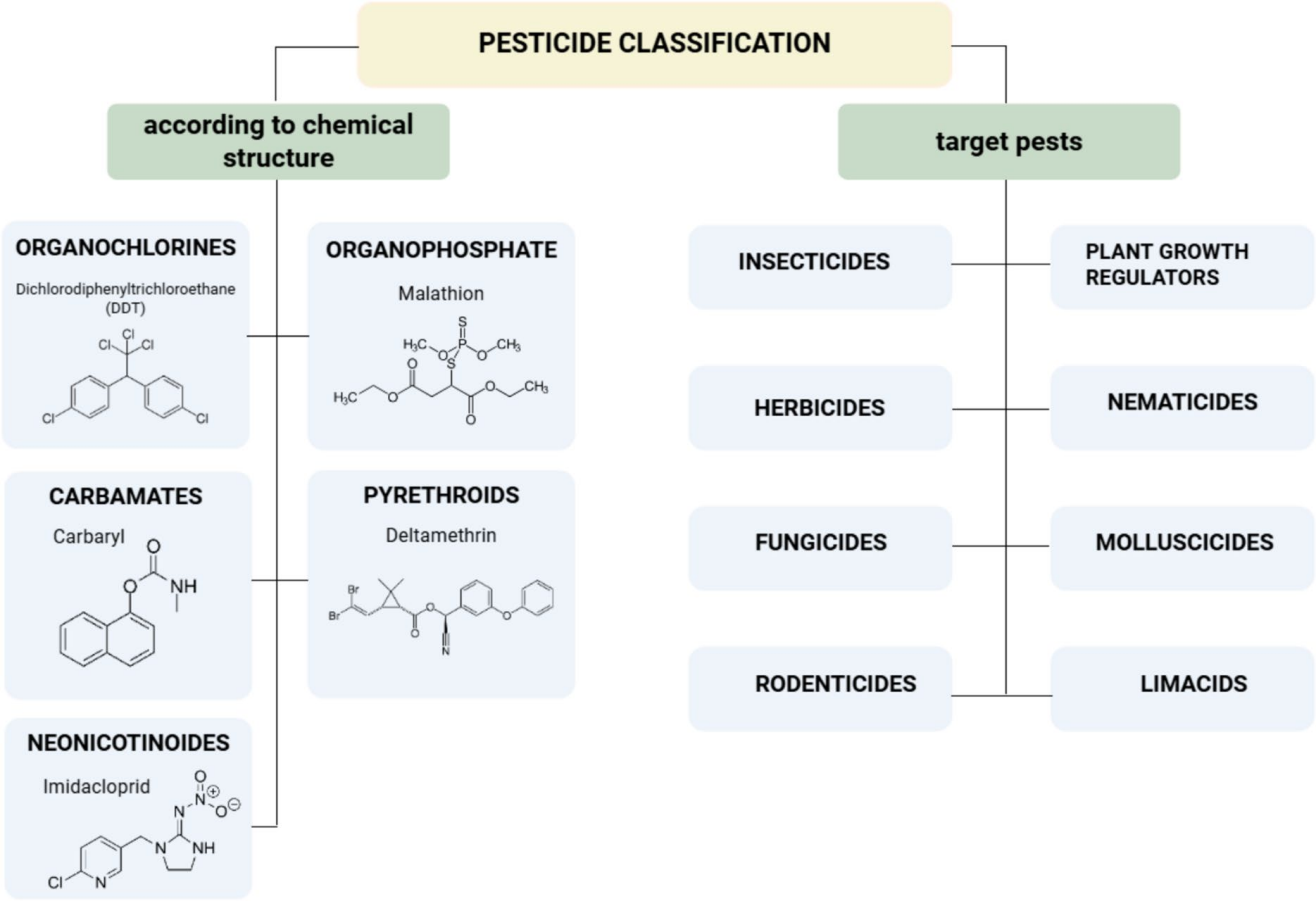
Classification of pesticides — the figure shows the classification of pesticides according to their purpose of action and chemical structure with selected representatives

As a society, we are exposed to a number of toxic substances, including pesticide exposure. Pesticides are a broad group of structurally different chemical substances with different biocidal properties. Based on the purpose of action, these substances are classified as insecticides, herbicides, fungicides, rodenticides, molluscicides, limacids, nematicides, and plant growth regulators (Ahamad et al., 2023; Garud et al., 2024; Hassaan & El Nemr, 2020). Due to the chemical structure, pesticides can be divided into organochlorine pesticides, organophosphates, carbamates, pyrethroids, and neonicotinoids — those are substances most commonly used in agriculture (Fig. 2) (Hakme et al., 2024; Umapathi et al., 2022). The mechanism of action of organophosphate pesticides involves the inhibition of acetylcholinesterase (AChE) by irreversible carbamylation of the enzyme, which leads to the accumulation of acetylcholine in cholinergic synapses and characteristic symptoms of poisoning (Park et al., 2009). Carbamates act similarly but cause reversible inhibition of AChE (Dorandeu et al., 2017). Neonicotinoids are classified as nicotinic acetylcholine receptor agonists (Mouskeftara et al., 2021).

### Routes of pesticide exposure

Some pesticides have been withdrawn from use due to their proven toxicity (e.g. dichlorodiphenyltrichloroethane (DDT)). Agricultural populations are constantly exposed to environmental xenobiotics, including a wide range of pesticides and other contaminants. Furthermore, a report issued by the World Health Organization (WHO) highlights that pesticide self-poisoning is one of the leading causes of suicide worldwide, causing over 150,000 deaths annually, mainly in low- and middle-income countries such as Sri Lanka, Bangladesh, and South Korea. Farm workers are more frequently exposed to pesticides due to improper storage and application procedures (Alcalá et al., 2024). Long–term exposure to low doses through inhalation and ingestion seems to be particularly dangerous (Anderson & Meade, 2014; Damalas & Eleftherohorinos, 2011; Damalas & Koutroubas, 2016; Shekhar et al., 2024). Dermal exposure is the most common route of exposure for farmers who use spraying (Tudi et al., 2022). Some of the absorbed xenobiotics are rapidly metabolized and excreted from the body, while others can bioaccumulate. Substances accumulating, among others, in the adipose tissue are responsible for toxic effects, including a number of endocrine disruptions (Lushchak et al., 2018; Treviño et al., 2023). Literature reports confirm the association between pesticide exposure and the occurrence of respiratory diseases and allergies (Ohlander et al., 2020). Control of pesticide exposure seems to be justified due to the potential health consequences among exposed individuals (Kim et al., 2017; Pascale & Laborde, 2020). However, these assessments mainly focus on single pesticides or within one class (J. Guo et al., 2020; Lazarevic et al., 2019; Li et al., 2023). This approach may not account for exposure to complex mixtures of pesticides commonly applied in agricultural settings.

### Determination of pesticides in standard biological materials

Current approaches and recent advances in analytical methods focus on the use of common biological matrices such as blood, urine, and breast milk, as well as hair and meconium (Crocoli et al., 2023; Yusa et al., 2015; Zuluaga et al., 2021). Organophosphates, organochlorines, and synthetic pyrethroids have been successfully identified in whole blood collected from Punjab residents (Bedi et al., 2015). Organochlorines and synthetic pyrethroids have also been identified in blood samples of residents of western Ethiopia (Afata et al., 2021). A study by Shalaby and Abdou (2020) demonstrated that farmers, pesticide vendors, and applicators living in Dakahlia Governorate in Egypt were exposed to highly toxic carbofuran and other pesticides such as chlorpyrifos and acetampiride. The high–performance liquid chromatography–mass spectrometry (UHPLC–MS/MS) analytical method developed by Matos et al. () allowed for the determination of fifteen pesticides from post–mortem blood samples, maintaining linearity in the range of 10–200 ng/ml. The method developed by Mouskeftara et al. (2021) used small sample volumes (50 μl) due to recognition of the realistic situation of impediment to large amounts of material collection. Despite the small volume of material, a simple, fast, and sensitive method for determination of nine pesticides in blood and urine was developed. Using the Quick–Easy–Cheap–Effective–Rrugged–Safe (QuEChERS) technique and gas chromatography, malathion, isofenphos–methyl, dichlorvos, chlorpyrifos, fentofil, p,p′–DDD, and p,p′–DDE were determined in human blood (Yu et al., 2017). In addition, a sensitive, fast method for determining 260 pesticides in urine using liquid chromatography coupled with mass spectrometer (LC–MS/MS) was optimized using the QuEChERS technique (Shin et al., 2019). In a study by Tarbah et al. (2001), a selective, one–step extraction of 23 organophosphate pesticides was developed in samples including urine, blood, and serum. Technological advances have led to the development of more sensitive detection methods that are able to identify pesticides in small quantities (C. W. Lee et al., 2016). The literature review confirms the high effectiveness of the liquid chromatography–mass spectrometry (LC/MS) technique in determining pesticides such as metaldehyde (Keighley et al., 2021; Schumacher et al., 2016; Szpot et al., 2018), betazone and its metabolites (Cho et al., 2017), imazalil and thiabendazole (Szpot & Buszewicz, 2013; Szpot et al., 2012), and glyphosate (Tsao et al., 2016), as well as paraquat and diquat (Tsao et al., 2016) in various materials. Mass spectrometry combined with chromatography remains one of the most selective, sensitive, and rapid analytical techniques for studying organic contaminants in various biological materials (Birolli et al., 2024).

### The use of DBS to identify pesticides — current state of knowledge

Standard whole blood and urine collection in the field poses certain difficulties. The preservation of blood in the form of dry spots is a relatively simple and minimally invasive method of material collection for identification of biomarkers of exposure to harmful factors (Jacobson et al., 2023). Literature reports confirm efficient extraction from DBS for two pesticides — fipronil (Raju et al., 2016) and paraquat (Wen et al., 2018). A study by Batterman and Chernyak (2014) indicated satisfactory extraction from DBS of organochlorine pesticides. Persistent organic pollutants are detectable in DBS in both children and adults (Seo & Batterman, 2024a). Based on poisoning statistics in Portugal, Soares et al. (2018) developed a method for identifying five common organophosphate insecticides in DBS. The presented method showed linearity in the range of 0.1–25 μg/ml for most compounds with detection limits from 0.05 to 0.1 μg/ml. Literature reports on the analysis of pesticides in DBS also focus on material collected from animals. A study by Provatas et al. () confirmed the successful gas chromatography–mass spectrometry (GC/MS) identification of polychlorinated biphenyls and organochlorine pesticides in the blood of marine mammals using a microsampling technique. Using LC–MS/MS, quantitative analysis of fipronil and its metabolites in DBS secured from rats and humans was possible (Raju et al., 2016). The analytical method and validation procedure conducted by Lehner et al. (2020) showed that, despite the small volume of DBS samples collected from birds, it was possible to identify chlorinated pesticides and polychlorinated biphenyls. Table 1 summarizes the recoveries for pesticides isolated from DBS based on isolation technique and spot volume — scientific publications focus mainly on organochlorine pesticides, omitting other chemicals used in agriculture. However, for a significant number of pesticides used in agriculture, there are no reports of isolation from DBS. The small amount of information on the isolation of pesticides, especially from DBS, emphasizes the need for the development of analytical methods that will enable the detection of these compounds in order to monitor pesticide exposure more thoroughly.

**Table 1:** Summary of different techniques for pesticides isolation from DBS — the extraction efficiency in the presented publications varies depending on the extract used and the sample volume

Authors	Method of detection	Extraction solvent	DBS sample volume (μl)	Pesticide	Recovery (%)
(Raju et al., 2016)	LC–MS/MS	Acetonitrile	10	Fipronil	95.72
				Fipronil sulfone	102.76
				Fipronil desulfinyl	89.51
(Soares et al., 2018)	GC–MS/M	Methanol:acetonitrile (50:50)	50	Diazinon	5.11
				Chlorpyrifos	1.19
				Ethylparathion	3.50
				Chlorfenwinphos	11.98
				Quinalfos	1.58
(Provatas et al., 2019) — organochlorine pesticide	GC–MS/MS	Methanol, formic acid	15	1,2,4,5–Tetrachlorobenzene	91.70
				1,2,3,4–Tetrachlorobenzene	107.20
				Pentachlorobenzene	107.80
				α–Hexachlorocyclohexane	90.50
				Hexachlorobenzene	96.50
				β–Hexachlorocyclohexane	81.60
				γ–Hexachlorocyclohexane	82.00
				Heptachlor	86.30
				Aldrin	105.30
				Heptachlor Epoxide	97.30
				Oxychlordane	65.30
				γ–Chlordane	74.60
				α–Chlordane	95.10
				α–Endosulfan	95.60
				trans–Nonachlor	65.20
				4,4′–Dichlorodifenylodichloroeten	101.80
				Dieldrin	88.20
				Endrin	84.30
				β–Endosulfan	92.30
				cis–Nonachlor	76.70
				4,4–Dichlorodiphenyldichloroethane	77.60
				4,4–Dichlorodiphenyltrichloroethane	95.70
				Photomirex	86.60
				Methoxychlor	86.00
				Mirex	72.10
(Lehner et al., 2020) — organochlorine pesticide	GC–MS/MS	Water, ethanol, decachlorobiphenyl in isooctane:ethanol (80:20), hexane:toluene (1:1)	50	α–Benzene Hexachloride	42.00
				γ–Benzene Hexachloride	63.00
				β–Benzene Hexachloride	124.90
				Δ–Benzene Hexachloride	124.90
				Heptachlor	77.60
				Aldrin	52.40
				Oxychlordane	74.20
				Heptachlor Epoxide	75.30
				trans–Chlordane	76.80
				trans–Nonachlor	62.30
				cis–Chlordane	62.80
				α–Endosulfan	75.50
				p,p′–Dichlorodiphenyldichloroethylene	71.50
				Dieldrin	73.20
				Endrin	77.50
				β–Endosulfan	110.80
				p,p′–Dichlorodiphenyldichloroethane	58.80
				p,p′–Dichlorodiphenyltrichloroethane	111.10
				Endrin aldehyde	1.50
				Methoxychlor	-
				Endosulfan sulfate	61.50
				Endrin ketone	-
(Batterman & Chernyak, 2014) — organochlorine pesticide	GC–MS	Hydrochloric acid, ethanol:isopropanol (1:1), hexane:methyl t–butyl ether (1:1), potassium chloride	50	Pentachlorobenzene	158.00
				Hexachlorobenzene	175.00
				Heptachlor	101.00
				cis/trans–Heptachlor Epoxide	-
				trans–Chlordane	120.00
				o,p′–Dichlorodiphenyldichloroethylene	100.00
				cis–Chlordane	106.00
				trans–Nonachlor	103.00
				p,p′–Dichlorodiphenyldichloroethylene	102.00
				cis–Nonachlor	103.00

## Conclusions

The microsampling technique and analysis of DBS have received increasing attention in clinical and forensic toxicology. This technique, due to its advantages, i.e. relatively easy material collection, ergonomic storage, and transport, may be a valuable complementary test for routine toxicological analysis in the future. The microsampling technique may be a practical way to assess exposure to pesticides, especially in rural areas or places where whole blood collection is difficult. Microsampling techniques seem to be especially beneficial in resource-limited regions with significant economic disparities, as they minimize costs related to sample collection, storage, and transport while enabling large-scale biomonitoring studies.

The subject still arouses much discussion due to impediment to the analysis of small spots of unknown volume, HCT effect, and the chromatographic effect. Difficulties with the estimation of the volume of the preserved spot constitute a key obstacle to the implementation of the technique in routine toxicological analyses. However, novel microsampling approaches such as VAMS have been developed to overcome these limitations by enabling the collection of fixed and accurate blood volumes independent of HCT, thereby improving analytical reliability. Despite the growing interest, there is still a limited number of scientific reports on the identification of pesticides in DBS. Toxicological analysis of DBS potentially may be a valuable assessment tool in pesticides’ exposure in the general population and occupationally exposed groups. The use of DBS may contribute to the expansion of the scale of pesticide biomonitoring, enabling the rapid and effective identification of exposed individuals. DBS analysis seems to be a practical tool for monitoring farmers’ exposure and supporting public health interventions. One of the significant limitations of pesticide determination in DBS is the small volume of available biological material, usually several microliters. Due to the fact that pesticides and their metabolites often occur in trace concentrations, the detection of these compounds requires the use of highly sensitive and selective analytical techniques. Additionally, a trace amount of analyte, secured in a small sample volume, increases the risk of errors related to the loss of analyte during extraction and the metric effect. Pesticides belong to a heterogeneous group of compounds with diverse physicochemical properties. The development of a universal extraction procedure for all classes may prove difficult. The extraction procedure of different pesticide classes may require the use of multi–stage extraction protocols, adapted to the physicochemical properties of the compounds being analysed; however, it increases the complexity of the analysis and the time of execution. Taking into account the advantages and disadvantages, the microsampling technique and the analysis of DBS require further research that will solve the most important analytical problems. In the context of the growing importance of assessment of the population exposure to toxic compounds, such as pesticides, the need for development and validation of analytical methods dedicated to this matrix seems particularly justified.

There is a significant research gap since the available scientific literature contains only a few reports on the determination of pesticides in DBS. However, the growing interest in DBS as an alternative and the fact of being a minimally invasive technique used for screening emphasize that the intensification of research in this area seems justified.

The body of literature on pesticide determination using dried blood spots remains limited, with relatively few publications addressing this topic. Consequently, some relevant studies may have been inadvertently overlooked due to language barriers. The small number of available studies restricts the ability to draw general conclusions about DBS analysis for pesticide biomonitoring. Heterogeneity in target analytes and analytical methods further complicates assessing the usefulness of dried blood spots analysis in toxicology. These points represent general limitations of the present review and of the studies it encompasses, highlighting the need for more comprehensive and standardized research in this field.

## Data Availability

No datasets were generated or analysed during the current study.
